# Supplementary material for: Adverse Childhood Experiences among Adults in North Carolina, USA: Influences on Risk Factors for Poor Health across the Lifespan and Intergenerational Implications
Source: Int J Environ Res Public Health. 2020 Nov 18;17(22):8548. doi: 10.3390/ijerph17228548 (PMC7698730; doi:10.3390/ijerph17228548)
Supplement: Supplementary file 1 [file ijerph-17-08548-s001.pdf]

Supplementary Table S1. Questions asked of respondents regarding Adverse Childhood Experiences (module included in 2012 & 2014). All questions refer to the time before you were 18 years of age. Now, looking back before you were 18 years of age—.

1. Did you live with anyone who was depressed, mentally ill, or suicidal?
   1 Yes
   2 No
   7 Don't know/Not sure
   9 Refused

2. Did you live with anyone who was a problem drinker or alcoholic?
   1 Yes
   2 No
   7 Don't know/Not sure
   9 Refused

3. Did you live with anyone who used illegal street drugs or who abused prescription medications?
   1 Yes
   2 No
   7 Don't know/Not sure
   9 Refused

4. Did you live with anyone who served time or was sentenced to serve time in a prison, jail, or other correctional facility?
   1 Yes
   2 No
   7 Don't know/Not sure
   9 Refused
5. Were your parents separated or divorced?
   1 Yes
   2 No
   8 Parents not married
   7 Don't know/Not sure
   9 Refused
6. How often did your parents or adults in your home ever slap, hit, kick, punch, or beat each other up?
   1 Never
   2 Once
   3 More than once
   7 Don't know/Not sure
   9 Refused
7. Before age 18, how often did a parent or adult in your home ever hit, beat, kick, or physically hurt you in any way? Do not include spanking. Would you say—
   1 Never
   2 Once
   3 More than once
   7 Don't know/Not sure
   9 Refused
8. How often did a parent or adult in your home ever swear at you, insult you, or put you down?
   1 Never
   2 Once
   3 More than once
   7 Don't know/Not sure
   9 Refused
9. How often did anyone at least 5 years older than you or an adult touch you sexually?

1 Never
2 Once
3 More than once
7 Don't know/Not sure
9 Refused

10. How often did anyone at least 5 years older than you or an adult, try to make you touch them sexually?
    1 Never
    2 Once
    3 More than once
    7 Don't know/Not sure
    9 Refused
11. How often did anyone at least 5 years older than you or an adult, force you to have sex?
    1 Never
    2 Once
    3 More than once
    7 Don't know/Not sure
    9 Refused

Supplementary Table S2. Prevalence of ACEs among included and excluded adults in North Carolina, 2012 & 2014.

ACE Category	Included (N=13,050)	Excluded (N=2,302)
	Weighted %	Weighted % (Cell N)
**Household Dysfunction**		
Household mental illness	15.2	20.7 (N=916)
Household alcohol abuse	22.7	35.9* (N=946)
Household substance use	10.7	15.1 (N=952)
Incarcerated family member	7.3	10.9* (N=974)
Parental separation or divorce	28.2	45.1* (N=930)
Any household dysfunction	48.1	66.6 (N=693)
**Emotional/Physical Abuse**		
Household physical violence	16.3	29.3* (N=733)
Physical abuse	13.9	25.0* (N=874)
Emotional abuse	28.1	39.2* (N=779)

Any emotional/physical abuse	35.8	53.4* (N=489)
**Sexual Abuse**		
Touched	9.4	12.2 (N=814)
Touched adult	7.0	8.0 (N=821)
Forced sex	4.3	6.2 (N=823)
Any sexual abuse	11.1	12.2 (N=716)
**Number of Adverse Childhood Experiences (ACEs)**		
0	40.4	-- (N=35)
1	23.5	-- (N=35)
2	11.2	-- (N=35)
3	8.3	-- (N=35)
4 or more	16.6	-- (N=35)

*Indicates statistically significant (p<0.01) difference between respondents who were included and excluded from the study. The number of excluded respondents varies since many people were excluded because they did not answer all ACE items. -- Estimate not reported because unweighted denominator <50.

Supplementary Table S3. Sensitivity analysis: Prevalence ratio (PR) of fair or poor health, current smoking, heavy alcohol consumption, overweight or obesity, frequent mental distress, and food insecurity among NC residents who answered at least 6 ACE items, 2012 & 2014.

Exposure Variables	Category	Fair or Poor General Health Status
		PR (95%CI)
**Within Appalachia (n=2.705)**		
**Number of ACEs**	**4-11**	1.05 (0.74-1.48)
	**0-3**	1.0 (reference group)
**Outside Appalachia (n=11,230)**		
Number of ACEs	4-11	1.65* (1.47-1.86)
	0-3	1.0 (reference group)

**Exposure Variables**	**Category**	**Outcomes**				
		Current Smoking[1] (n=13,912)	Heavy Alcohol[1] (n=13,546)	Over- weight or Obese[1] (n=13,101)	Frequent Mental Distress[1] (n=13,763)	Food Insecurity[1,2] (n=10,867)
		PR (95% CI)	PR (95% CI)	PR (95% CI)	PR (95% CI)	PR (95% CI)
Number of ACEs	4-11	1.56* (1.37-1.78)	1.69§ (1.25-2.27)	1.07§ (1.02-1.12)	2.45* (2.07-2.89)	1.58* (1.45-1.74)
	0-3	1.0 (reference group)				
Region of Residence	In Appalachia	1.10 (0.94-1.30)	1.09 (0.80-1.49)	0.99 (0.96-1.04)	0.97 (0.78-1.20)	1.13† (1.02-1.25)
	Outside Appalachia	1.0 (reference group)				

Note: There was a statistically significant interaction between Appalachian residence and ACEs for the fair or poor health status outcome, and therefore separate models were run for within and outside Appalachia. All other models had no significant interaction, so we included Appalachian residence as a covariate. [1]Adjusted for age, age squared, sex, and education category [2]Uses 2012 BRFSS data only *p-value <0.001 §p-value <0.01. †p-value <0.05.

© 2020 by the authors. Licensee MDPI, Basel, Switzerland. This article is an open access article distributed under the terms and conditions of the Creative Commons Attribution (CC BY) license (http://creativecommons.org/licenses/by/4.0/).
